# Serial ASPECTS to predict stroke-associated pneumonia after thrombolysis in patients with acute ischemic stroke

**DOI:** 10.3389/fneur.2024.1364125

**Published:** 2024-04-22

**Authors:** Sarawut Krongsut, Atiwat Soontornpun, Niyada Anusasnee

**Affiliations:** ^1^Division of Neurology, Department of Internal Medicine, Faculty of Medicine, Saraburi Hospital, Saraburi, Thailand; ^2^Division of Neurology, Department of Internal Medicine, Faculty of Medicine, Chiang Mai University, Chiang Mai, Thailand; ^3^Division of Radiology, Saraburi Hospital, Saraburi, Thailand

**Keywords:** ischemic stroke, cerebrovascular disease, thrombolysis, ASPECTS, stroke-associated pneumonia

## Abstract

**Background:**

Stroke-associated pneumonia (SAP) is a serious complication in stroke patients, significantly increasing mortality. The Alberta Stroke Program Early CT Score (ASPECTS) is a recognized predictor of acute ischemic stroke outcomes. We aimed to investigate the performance of serial ASPECTS assessments (baseline ASPECTS, 24-h ASPECTS, and change in ASPECTS) for predicting SAP in patients with thrombolyzed acute anterior circulation ischemic stroke (AACIS).

**Materials:**

A retrospective observational cohort study of adult patients with thrombolyzed AACIS was conducted. Baseline and 24-h ASPECTS using non-contrast computed tomography (NCCT), complications of stroke, including SAP and swallowing dysfunction using the Modified Water Swallowing test, were collected. Baseline and 24-h ASPECTS were evaluated by a certified neurologist and neuroradiologist. The predictive performance was determined based on the receiver operating characteristic curve (ROC). Multivariable logistic regression analyses were employed to assess the impact of serial ASPECTS assessment on predicting SAP.

**Results:**

Of the 345 patients with thrombolyzed AACIS in our study, 18.4% (64/345) experienced SAP. The patients’ median age was 62 years [interquartile range (IQR): 52–73], with 53.4% being male. The median NIHSS score was 11 points (IQR: 8–17). The ROC analysis revealed areas under the curve for predicting SAP with baseline ASPECTS, 24-h ASPECTS, and change in ASPECTS were 0.75 (95% CI, 0.69–0.82), 0.84 (95% CI, 0.79–0.89), and 0.82 (95% CI, 0.76–0.87), respectively. Of the three measures, 24-h ASPECTS was a better predictor of SAP (odds ratio: 5.33, 95%CI: 2.08–13.67, *p* < 0.001) and had a higher sensitivity (0.84 [95%CI, 0.74–0.92]) and specificity (0.79 [95%CI, 0.74–0.84]) than both baseline ASPECTS and change in ASPECTS.

**Conclusion:**

24-h NCCT-ASPECTS outperformed both baseline ASPECTS and change in ASPECTS for predicting SAP. Notably, 24-h ASPECTS, with a cut-off value of ≤6, exhibited good predictive performance and emerged as the better predictor for SAP.

## 1 Introduction

Stroke represents a substantial global public health challenge, being a leading cause of both mortality and disability (1). In Thailand, it ranks as the primary cause of death for females and the third most common for males, with a mortality rate of 10% and a 50% prevalence of disabilities among survivors (1). The incidence of ischemic stroke in Thailand is increasing as the average age of the population continues to rise. Intravenous recombinant tissue plasminogen activator (IV-tPA) is the standard treatment for patients with acute ischemic stroke (AIS) (2). Complications after stroke are associated with increased mortality and length of hospital stay. Most of these complications appear within the first week after AIS (3). Disability and mortality increase with the number of complications, particularly stroke-associated pneumonia (SAP) and other infections (4). SAP is a commonly encountered complication in clinical practice. The incidence of SAP as reported in previous studies varies depending on the hospital treatment facility, ranging from 10% to 57% for patients treated in intensive care units to 4%–12% for patients treated in stroke units (4, 5). This demonstrates that it is crucial to prioritize early detection and thorough monitoring of patients at high-risk for SAP. This proactive approach is designed to support physicians in treatment decision-making and ultimately to lead to reduced mortality rates and improved prognosis among patients with AIS receiving thrombolytic therapy.

Due to the lack of specific clinical symptoms and neuro-radiographic information that can aid in the early prediction of SAP, the appearance of complications often indicates a severe SAP that frequently results in mortality. In response to that situation, development of a prediction model was undertaken to identify patients at high risk for SAP (6, 7). Known risk factors for SAP include older age, male gender, diabetes, hypertension, atrial fibrillation (AF), congestive heart failure (CHF), chronic obstructive pulmonary disease (COPD), pre-existing dependency, various stroke subtypes, dysphagia, and greater stroke severity (8). The Alberta Stroke Program Early CT Score (ASPECTS) is a 10-point quantitative topographic CT scan score in patients with middle cerebral artery (MCA) stroke to assess early ischemic changes and cytotoxic edema (9). A previous study reported that ASPECTS and the National Institute of Health Stroke Score (NIHSS) had a strong negative correlation coefficient of −0.680 (*p* < 0.001) (10). Previous studies have also shown that 24-h NIHSS can predict long-term stroke outcomes more accurately than change in NIHSS and baseline NIHSS in patients treated with IV-tPA and mechanical thrombectomy (11, 12). Patients receiving IV-tPA typically undergo a 24-h follow-up using non-contrast computed tomography (NCCT). This period before the follow-up is of clinical significance as it provides a window of opportunity for re-evaluation of brain NCCT to exclude the presence of hemorrhagic transformation, a condition which is closely associated with stroke prognosis and which aids in assessing the size and location of cerebral ischemia. Thus, the assessment of serial ASPECTS (baseline ASPECTS, 24-h ASPECTS, and change ASPECTS) could serve as a crucial predictor for SAP in patients with AIS undergoing thrombolytic treatment.

Several studies have generally reported that ASPECTS was a useful tool in predicting functional outcome and intracerebral hemorrhage (ICH) (13, 14). In light of the current insufficiency of and lack of clarity in the available data including the lack of knowledge regarding the prognostic value of serial ASPECTS assessment, 24-h ASPECTS assessment could potentially serve as an important tool in predicting SAP. To investigate that possibility, the present study was designed to compare the prognostic value and assess the potential impact of baseline ASPECTS, 24-h ASPECTS, and change in ASPECTS on NCCT for predicting SAP in patients with thrombolyzed acute anterior circulation ischemic stroke (AACIS).

## 2 Materials and methods

### 2.1 Study population

**Figure 1:**
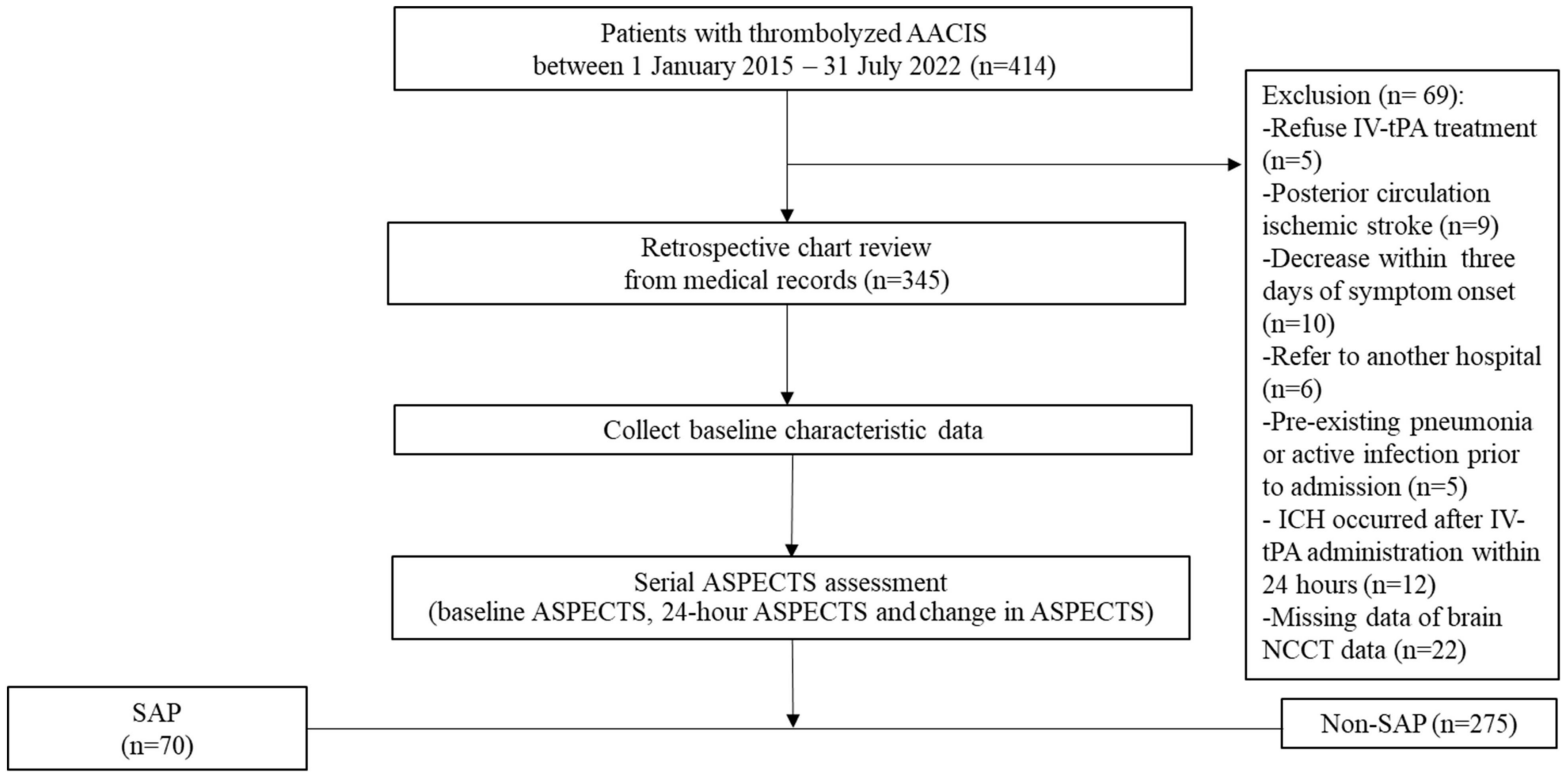
Study flow diagram.

We retrospectively analyzed the clinical and brain NCCT data of consecutively 345 patients with thrombolyzed AACIS who were admitted to the stroke unit at Saraburi Hospital, a provincial hospital in Thailand with 700 in-patient beds, between 1 January 2015 and 31 July 2022. This protocol is aligned with the standards of “Strengthening the Reporting of Observational Studies in Epidemiology” (15); a detailed checklist is available in Supplementary Table 1. All enrolled patients in this study are admitted to a stroke unit. During the Coronavirus Disease 2019 (COVID-19) pandemic, every patient admitted to the stroke unit must demonstrate no evidence of COVID-19 infection, as confirmed by polymerase chain reaction testing for COVID-19 prior to admission. Patients who receive early tracheal intubation will be transferred to the intensive care unit for treatment. Approval for this study was obtained from the institutional review board of Saraburi Hospital on 2 May 2023. We accessed the data for research purposes on 28 May 2023. All patients with AACIS were treated according to the guidelines for the early management of patients with AIS 2019 (16). The patient flow chart is shown in Figure 1. The inclusion criteria were as follows: (i) age ≥ 18 years; (ii) patients diagnosed with AACIS and who received IV-tPA. The exclusion criteria were as follows: (i) pregnancy; (ii) patients with posterior circulation ischemic stroke; (iii) patients deceased within 3 days of symptom onset; (iv) patients referred to other hospitals whose treatment information could not be followed up; (v) pre-existing pneumonia, active infection prior to admission, or previous antibiotic treatment; (vi) ICH occurred after IV-tPA administration within 24 h (vii) patients with missing data, such as NIHSS, NCCT images, and laboratory results; and (viii) patients who received endovascular treatment (EVT). During this study period, EVT could not be performed in our center, and patients with large vessel occlusions could not be referred for further treatment. This was due to healthcare policy restrictions regarding reimbursement for this treatment in Thailand. All Patients or their families provided consent for IV-tPA through signed informed consent forms. Study patients received IV-tPA at 0.9 mg/kg, administered as a 10% bolus followed by the remainder over 1 h. Blood pressure was maintained below 180/105 mmHg during thrombolysis and the subsequent 24 h.

### 2.2 Data collection

We reviewed the electronic medical records for demographic and clinical characteristics including age, gender, comorbidities (prior ischemic stroke, AF, myocardial infarction (MI), CHF, COPD, valvular heart disease, diabetes, hypertension, chronic kidney disease (CKD) and history of renal replacement therapy, dyslipidemia, history of malignancy), active tobacco smoking, active alcohol consumption, pre-stroke functional status using the modified Rankin Scale (mRS), clinical presentation, NIHSS, laboratory data [white blood cell (WBC), neutrophil-to-lymphocyte ratio (NLR), hemoglobin, hematocrit, platelet, INR, creatinine, and fasting plasma glucose (FPG)], NCCT-ASPECTS at baseline and at 24-h follow-up, and complications of stroke including SAP. AIS was identified based on the International Classification of Diseases, Tenth Revision, and Clinical Modification codes with a diagnostic code of I63 (17).

ASPECTS provides CT scan reliability using a grading system to assess early ischemic changes. It quantifies these changes in hyperacute ischemic stroke within the MCA territory, divided into 10 regions. Points are deducted for regions with early ischemic signs. The ASPECTS score was designed to identify patients who would benefit most from thrombolysis and/or thrombectomy (18). An NCCT scan was obtained at admission for all patients. CT scans were repeated after 24-h in those patients who had received IV-tPA in order to evaluate the infarct area and to identify hemorrhagic transformation. Baseline ASPECTS was defined as the NCCT-ASPECTS prior to IV-tPA. 24-h ASPECTS was defined as NCCT-ASPECTS at 24 h after IV-tPA. Change in ASPECTS was defined as the baseline ASPECTS minus 24-h ASPECTS.

The scoring system for baseline and 24-h ASPECTS uses the same system. NCCT brain images were evaluated by a certificated neurologist and neuroradiologist. Individuals performing the imaging were blinded to the patients’ clinical information. In cases where discrepancies in ASPECTS values arose, resolution was achieved through collaborative discussions between the neurologist and neuroradiologist to ensure accurate ASPECTS results. The entire cranial region from the base of the skull to the vertex was evaluated using a TOSHIBA-160 slice scanner (Aquilion Prime, Canon Medical Systems, Otawara, Japan) at Saraburi Hospital. Continuous cross-sectional images were acquired parallel to the inferior orbitomeatal line, with a slice thickness of 3 mm, utilizing 120 kV and 240-mA interpretation settings.

Swallowing dysfunction was assessed using the Modified Water Swallowing Test (MWST) (19). Patients were screened for dysphagia within 24 h of admission. At our stroke center, all patients with AIS undergo a comprehensive clinical swallowing examination conducted by stroke-trained nursing staff and occupational therapists. The patients are seated in the upright position and swallow 3 mL of water three times. The results of each swallowing are recorded on a 5-point scale as follows: (1) unable to swallow, (2) swallowing with abnormal breathing, (3) swallowing with voice change or choking, (4) swallowing without choking or respiratory change, and (5) swallowing normally and able to repeat swallowing at least twice in 30 s. Scoring was done each time the patient swallowed and the lowest score of the three swallowings was used to quantify the severity of the dysphagia. A score of less than 4 indicated swallowing dysfunction. This evaluation included a clinical assessment of consciousness, oral motor skills, articulating function, and MWST. A nasogastric tube was inserted in all patients with comatose or swallowing dysfunction to prevent aspiration. Protocols related to research articles are available on the protocols.io platform, accessible through this link: https://dx.doi.org/10.17504/protocols.io.q26g7p6qkgwz/v1.

### 2.3 Outcomes

SAP was diagnosed based on the modified Centers for Disease Control and Prevention criteria (20) and/or Mann’s criteria (21). It was defined as pneumonia complicating in the first 7 days after stroke onset in nonventilated patients, with consideration of clinical symptoms, laboratory evidence of respiratory infection, positive radiologic findings, and/or bacterial culture results from respiratory specimens. Patients meeting the definitive criteria for SAP were classified as having SAP.”

### 2.4 Statistical analysis

Statistical analysis was performed using Stata software version 16.0 (StataCorp, College Station, TX, United States). Continuous variables are presented as median and interquartile range (IQR), while categorical variables were reported as count and percentage. Differences in continuous variables between SAP and non-SAP patients were assessed using Student’s *t*-test or the nonparametric Mann–Whitney U-test, and χ2 or Fisher’s exact test were used for categorical variables. Receiver operating characteristic (ROC) curves were generated using binary logistic regression to compare the predictive performance of baseline ASPECTS, 24-h ASPECTS, and change in ASPECTS for SAP prediction. The area under the ROC curve (AuROC) was compared using nonparametric methods (22), and the optimal cut-off value was determined using the Youden’s index method. Relationships between serial ASPECTS evaluations and the risk of SAP were estimated using univariable analysis and binary logistic regression analyses. We adjusted for all potential confounders with *p*-value less than 0.2 in univariable analysis, including age, sex, AF, MI, CHF, COPD, CKD, history of malignancy, swallowing dysfunction, preexisting disability, systolic blood pressure, diastolic blood pressure, NIHSS at admission, WBC, NLR, creatinine, and FPG. These variables were utilized in the multivariate analysis to conduct logistic regression to determine the independent determinants of SAP. Separate multivariate analyses were conducted to assess the impact of baseline ASPECTS, 24-h ASPECTS, and change in ASPECTS on SAP prediction and to address the issue of multicollinearity among the variables. The model’s fit was evaluated using the Hosmer-Lemeshow Goodness of Fit statistic, and *p*-value greater than 0.05 suggested a satisfactory fit. Correlations are expressed as odds ratios (ORs) with 95% confidence intervals (CI). We considered two-sided *p*-values of <0.05 to be statistically. Additionally, correlation analysis was used to explore the relationship between 24-h ASPECTS, predictive factors and biomarkers of inflammatory response in the patient cohort.

## 3 Results

Overall, 414 consecutive AIS patients who meet the eligibility criteria of IV-tPA were enrolled. Of these, 69 were excluded due to refusal to undergo IV-tPA (*n* = 5), posterior circulation ischemic stroke (*n* = 9), decease within 3 days of symptom onset (*n* = 10), referral to another hospital (*n* = 6), pre-existing pneumonia or active infection prior to admission (*n* = 5), ICH occurred after IV-tPA administration within 24 h (*n* = 12), and missing brain NCCT data (*n* = 22). Finally, a total of 345 patients were included in the analysis (Figure 1).

### 3.1 Comparison of baseline clinical characteristics between the two groups

Overall prevalence of SAP was 18.4% (64/345). The patients’ median age was 62 years (IQR: 52–73), 53.4% were male, the mean follow-up time was 8.76 ± 10.7 days, and the median NIHSS score was 11 points (IQR: 8–17 points). The patients with SAP were more likely to be older, have poorer pre-stroke functional status, higher diastolic blood pressure, higher NIHSS scores, and a higher likelihood of having AF, CHF, CKD, swallowing dysfunction, aphasia, and/or gaze paresis compared to those without SAP (all *p* < 0.05). No statistically significant differences were observed for gender, time to treatment with thrombolysis, history of prior stroke, diabetes, or dyslipidemia (Table 1).

**Table 1:** Comparison of demographic, clinical, and laboratory findings between SAP and non-SAP.

Characteristic	All patients (*n* = 345)	SAP (*n* = 70)	Non-SAP (*n* = 275)	*P*-value
Age (years)—median (IQR)	62 (52–73)	68 (58–80)	60 (50–71)	<0.001
**Gender, *n* (%)**				
Male	183 (53.0)	38 (54.3)	145 (52.7)	0.816
Female	162 (47.0)	32 (45.7)	130 (47.3)	
**Vascular risk factor and comorbidities, *n* (%)**				
Smoking	123 (35.7)	21 (30.)	102 (37.1)	0.269
Alcohol	142 (41.2)	28 (40.0)	114 (41.5)	0.825
Prior stroke	41 (11.9)	8 (11.4)	33 (12.0)	0.895
Atrial fibrillation	102 (29.6)	35 (50.0)	67 (24.4)	<0.001
Myocardial infarction	29 (8.4)	9 (12.9)	20 (7.3)	0.133
Congestive heart failure	37 (10.7)	16 (22.9)	21 (7.6)	<0.001
Valvular heart disease	22 (6.4)	5 (7.1)	17 (6.2)	0.769
COPD	10 (2.9)	4 (5.71)	6 (2.18)	0.123
Diabetes mellitus	93 (27.0)	21 (30.0)	72 (26.2)	0.520
Hypertension	243 (70.4)	52 (74.3)	191 (69.5)	0.429
Chronic kidney disease	44 (12.8)	15 (21.4)	29 (10.6)	0.015
Dyslipidemia	141 (40.9)	28 (40.0)	113 (41.1)	0.868
Gout	11 (3.2)	4 (5.7)	7 (2.6)	0.244
History of malignancy	8 (2.3)	4 (5.7)	4 (1.5)	0.057
History of renal replacement therapy	5 (1.5)	2 (2.9)	3 (1.1)	0.268
**Clinical presentation, *n* (%)**				
Hemiparesis	341 (98.8)	70 (100.0)	271 (98.6)	0.586
Dysarthria	275 (79.7)	59 (84.3)	216 (78.6)	0.322
Swallowing dysfunction (MWST < 4)	129 (37.4)	62 (88.6)	67 (24.4)	<0.001
Ataxia	37 (10.7)	12 (17.1)	25 (9.1)	0.052
Hemianopia	23 (6.7)	8 (11.4)	15 (5.5)	0.074
Aphasia	132 (38.3)	47 (67.1)	85 (30.9)	<0.001
Neglect	62 (18.0)	18 (25.7)	44 (16.0)	0.059
Cranial nerve disorder	12 (3.5)	3 (4.3)	9 (3.3)	0.715
Gaze paresis	112 (32.5)	49 (70.0)	63 (22.9)	<0.001
**Pre-stroke functional status, *n* (%)**				
Preexisting disability (mRS)				<0.001
0	320 (92.8)	54 (77.1)	266 (96.7)	
1	6 (1.7)	2 (2.9)	4 (1.5)	
2	17 (4.9)	13 (18.6)	4 (1.5)	
3	2 (0.6)	1 (1.4)	1 (0.3)	
**Time to rt-PA, hours**				
<3 h	233 (67.5)	46 (65.7)	187 (68.0)	0.715
3–4.5 h	112 (32.5)	24 (34.3)	88 (32.0)	
SBP (mmHg)—median (IQR)	154 (140–178)	163 (142–190)	153 (140–173)	0.095
DBP (mmHg)—median (IQR)	90 (80–101)	95 (81–108)	90 (80–100)	0.041
**NIHSS at admission, *n* (%)**				
4–15	232 (67.3)	17 (24.3)	215 (78.2)	<0.001
16–20	76 (22.0)	33 (47.1)	43 (15.6)	
>20	37 (10.7)	20 (28.6)	17 (6.2)	
Hospital stays (days)—median (IQR)	5 (3–9)	13 (7–23)	4 (3–7)	<0.001
**Laboratory**				
WBC (x10^3^/μL) median (IQR)	8.50 (7.00–10.30)	9.50 (7.50–11.90)	8.40 (7.00–10.00)	0.006
NLR—median (IQR)	2.2 (1.5–3.7)	2.7 (1.6–5.3)	2.2 (1.5–3.6)	0.053
Hb (g/dL)—median (IQR)	12.7 (11.3–14.0)	12.4 (10.6–14.0)	12.7 (11.5–14.0)	0.432
Hct (%)—median (IQR)	38 (35–42)	38 (33–42)	39 (35–42)	0.437
Platelet (x10^3^/μL)—median (IQR)	243 (203–289)	237 (204–284)	244 (203–290)	0.878
INR—median (IQR)	0.96 (0.90–1.00)	0.98 (0.90–1.01)	0.95 (0.90–1.00)	0.244
Cr (mg/dL)—median (IQR)	0.95 (0.78–1.15)	1.00 (0.81–1.24)	0.93 (0.77–1.13)	0.049
FPG (mg/dL)—median (IQR)	112 (95–141)	124 (106–145)	106 (93–136)	0.001
**Workflow time**				
Onset to door (minutes)—median (IQR)	90 (60–120)	90 (60–120)	90 (60–120)	0.967
Door to needle time (minutes)—median (IQR)	42 (28–63)	47 (30–68)	41 (27–62)	0.509
Onset-to-baseline CT time (minutes)—median (IQR)	114 (77–146)	116 (78–149)	112 (76–144)	0.725
**ASPECTS, median (IQR)**				
Baseline ASPECTS	10 (8–10)	7 (6–9)	10 (9–10)	<0.001
24-h ASPECTS	8 (5–9)	4 (2–6)	9 (7–10)	<0.001
Change in ASPECTS	1 (0–3)	4 (2–5)	1 (0–2)	<0.001
**Complications, *n* (%)**				
Urinary tract infection	31 (9.0)	13 (18.6)	18 (6.6)	0.002
sICH	42 (12.2)	26 (37.1)	16 (5.8)	<0.001
Progressive stroke	58 (16.8)	27 (38.6)	31 (11.3)	<0.001
Seizure	12 (3.5)	5 (7.1)	7 (2.6)	0.061
Septicemia	24 (7.0)	18 (25.7)	6 (2.2)	<0.001
Brain herniation	27 (7.8)	12 (17.1)	15 (5.5)	0.001
Craniectomy	15 (4.4)	9 (12.9)	6 (2.2)	<0.001
Mechanical ventilator requirement^*^	67 (19.4)	46 (65.7)	21 (7.6)	<0.001

ASPECTS, Alberta stroke program early CT score; Cr, creatinine; COPD, chronic obstructive pulmonary disease; CT, computed tomography; DBP, diastolic blood pressure; FPG, fasting plasma glucose; Hb, hemoglobin; Hct, hematocrit; IQR, interquartile range; mRS, modified Rankin Scale; MWST, Modified Water Swallowing Test; NIHSS, the National Institutes of Health Stroke Scale; NLR, neutrophil to lymphocyte ratio; SAP, stroke associated pneumonia; SBP, systolic blood pressure; sICH, symptomatic intracerebral hemorrhage (hemorrhagic transformation after thrombolysis); WBC, white blood cell. ^*^Event occurred after SAP.

### 3.2 Comparison of laboratory results and complications between the two groups

Patients with SAP had higher WBC, creatinine, and FPG compared to those without SAP (all *p* < 0.05). There were no significant differences in NLR, hemoglobin, hematocrit, platelet count, or INR levels between the two groups. Additionally, patients with SAP had a significantly higher incidence of stroke complications, with the exception of seizure, compared to those without SAP (Table 1).

### 3.3 Distribution of ASPECTS

Baseline ASPECTS and 24-h ASPECTS of patients with SAP were lower than those without SAP (7 [IQR, 6–9] vs. 10 [IQR, 9–10] and 4 [IQR, 2–6] vs. 9 [IQR, 7–10], respectively, *p* < 0.001). In addition, patients with SAP had a greater change in ASPECTS (4 [IQR, 2–5] vs. 1 [IQR, 0–2], *p* < 0.001; Table 1). Significant differences were observed in the distribution of serial ASPECTS assessments between patients with and without SAP (*p* < 0.001). The SAP rates in patients with baseline ASPECTS ≤8, 24-h ASPECTS ≤ 6, and change in ASPECTS ≥ 2 were 68.6% (48/70), 84.3% (59/70), and 81.4% (57/70), respectively (Table 2).

**Table 2:** Distribution of ASPECTS in patients with SAP after thrombolysis for patients with AACIS.

ASPECTS	SAP (*n* = 70)	Non-SAP (*n* = 275)	*P*-value
**Baseline ASPECTS**			
≤8	48 (68.6)	61 (22.2)	<0.001
>8	22 (31.4)	214 (77.8)	
**24-h ASPECTS**			
≤6	59 (84.3)	58 (21.1)	<0.001
>6	11 (15.7)	217 (78.9)	
**Change in ASPECTS**			
<2	13 (18.6)	182 (66.2)	<0.001
≥2	57 (81.4)	93 (33.8)	

ASPECTS, Alberta stroke program early CT score; AACIS, acute anterior circulation ischemic stroke; SAP, stroke associated pneumonia.

### 3.4 Discriminative accuracy of scores in predicting SAP

**Figure 2:**
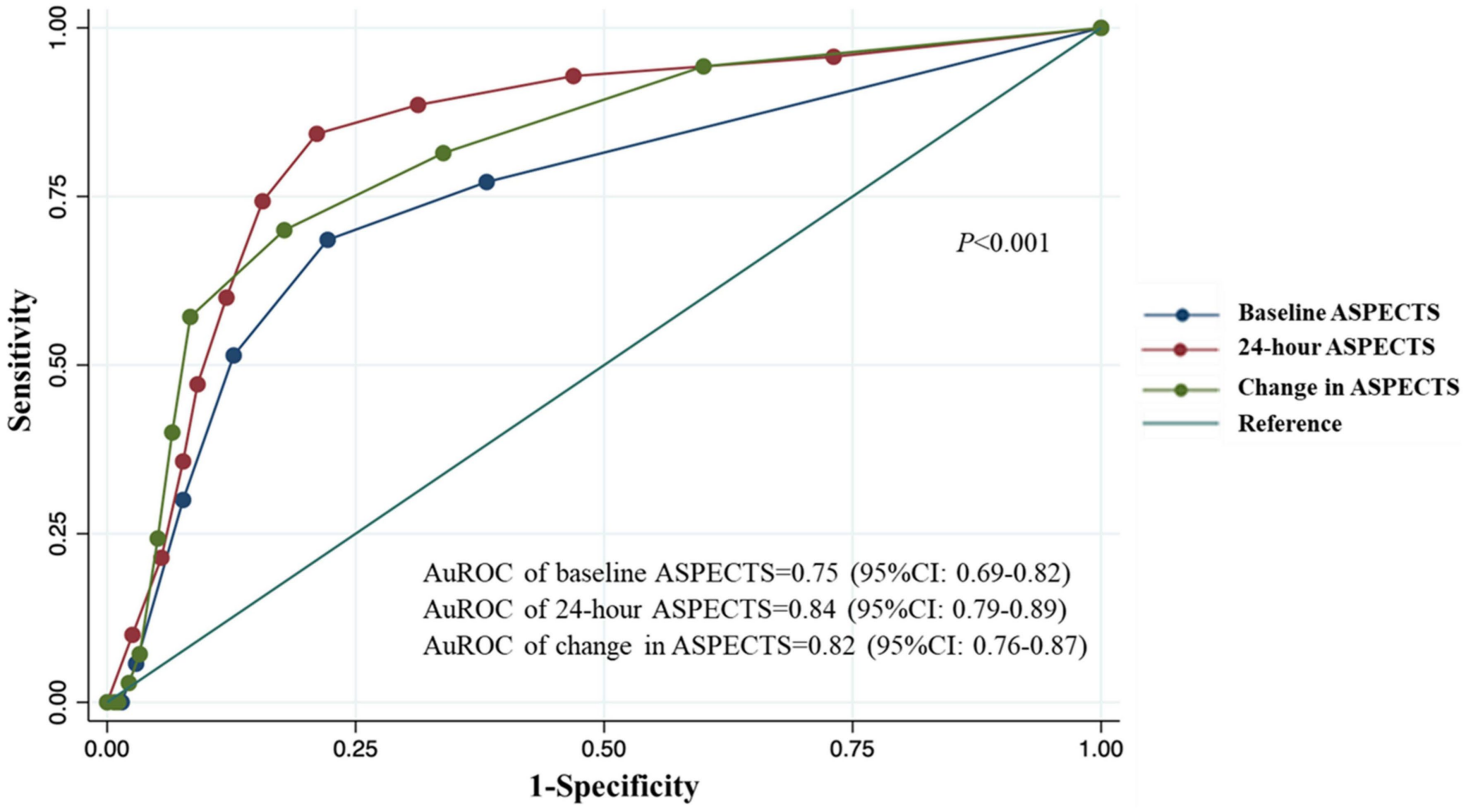
ROC and AuROC for predicting SAP after thrombolysis in patients with acute ischemic stroke.

From the ROC analysis, we found that 24-h ASPECTS showed a higher sensitivity and specificity than both baseline and change in ASPECTS. The optimal cut-off value was ≤6 for 24-h ASPECTS, whose sensitivity was 84.3% (95%CI; 73.6–91.9), specificity was 78.9% (95%CI; 73.6–83.6), positive predictive value was 50.4% (95%CI; 41.0–59.8), and negative predictive value was 95.2% (95%CI; 91.5–97.6). Baseline ASPECTS showed the lowest sensitivity for predicting SAP. The highest Youden’s index was found at the cut-off value of ≤8 in baseline ASPECTS (0.464), ≤6 in 24-h ASPECTS (0.632), and ≥2 in change in ASPECTS (0.522). The ROC curves of a serial ASPECTS assessment are shown in Figure 2. The AuROC of baseline ASPECTS, 24–hour ASPECTS, and change in ASPECTS for predicting SAP were 0.75 (95% CI, 0.69–0.82), 0.84 (95%CI, 0.79–0.89), and 0.82 (95%CI, 0.76–0.87), respectively (Table 3). The box-plots of serial ASPECTS by SAP are shown in Supplementary Figure 1.

**Table 3:** The optimal cut-off score and the predictive values of ASPECTS to predict SAP.

Variable	AuROC	Optimal cut-off	Sensitivity (%)	Specificity (%)	PPV (%)	NPV (%)	LR+	LR−	Accuracy (%)
	(95%CI)		(95%CI)	(95%CI)	(95%CI)	(95%CI)	(95%CI)	(95%CI)	(95%CI)
Baseline ASPECTS	0.75 (0.69–0.82)	≤8	68.6 (56.4–79.1)	77.8 (72.4–82.6)	44.0 (34.5–53.9)	90.7 (86.2–94.1)	3.09 (2.35–4.06)	0.41 (0.28–0.57)	75.9 (71.1–80.4)
24-h ASPECTS	0.84 (0.79–0.89)	≤6	84.3 (73.6–91.9)	78.9 (73.6–83.6)	50.4 (41.0–59.8)	95.2 (91.5–97.6)	4.00 (3.11–5.13)	0.20 (0.12–0.34)	80.0 (75.4–84.1)
Change in ASPECTS	0.82 (0.76–0.87)	≥2	81.4 (70.3–89.7)	66.2 (60.3–71.8)	38.0 (30.2–46.3)	93.3 (88.9–96.4)	2.41 (1.97–2.94)	0.28 (0.17–0.46)	69.3 (64.1–74.1)

ASPECTS, Alberta Stroke Program Early CT Score; AuROC, area under the receiver operating characteristic; CI, confidence interval; LR+, the positive likelihood ratio; LR−, the negative likelihood ratio; NPV, negative predictive value; PPV, positive predictive value; SAP, stroke associated pneumonia.

### 3.5 Association between serial ASPECTS assessment and SAP

From the multivariable logistic regression analysis (MVLR), the adjusted ORs were 2.09 (95% CI, 0.95–4.61; *p* = 0.067), 5.33 (95% CI, 2.08–13.67; *p* < 0.001), and 3.54 (95% CI, 1.43–8.77; *p* = 0.006) for baseline ASPECTS ≤8, 24-h ASPECTS ≤ 6, and change in ASPECTS ≥2, respectively. 24-h ASPECTS of ≤6 and change in ASPECTS ≥ 2 were significantly associated with SAP (Table 4). The non-significant Hosmer-Lemeshow test (*p* = 0.644 for baseline ASPECTS model, *p* = 0.976 for 24-h ASPECTS model, and *p* = 0.962 for change in ASPECTS model), indicated a good fit to the model.

**Table 4:** Association between serial ASPECTS assessment and SAP after thrombolysis.

Predictors	Univariable analysis		Multivariable analysis					
	OR (95%CI)	*P*-value	Baseline ASPECTS		24-h ASPECTS		Change in ASPECTS	
			aOR (95%CI)	*P*-value	aOR (95%CI)	*P*-value	aOR (95%CI)	*P*-value
**Main exposure**								
Baseline ASPECTS ≤ 8	7.65 (4.29, 13.66)	<0.001	2.09 (0.95, 4.61)	0.067	NA	NA	NA	NA
24-h ASPECTS ≤ 6	20.07 (9.91, 40.65)	<0.001	NA	NA	5.33 (2.08, 13.67)	<0.001	NA	NA
Change in ASPECTS ≥ 2	8.58 (4.47, 16.47)	<0.001	NA	NA	NA	NA	3.54 (1.43, 8.77)	0.006
**Covariates**								
Age (per 1 year older)	1.04 (1.02, 1.06)	<0.001	1.03 (1.00, 1.07)	0.030	1.04 (1.01, 1.07)	0.022	1.03 (1.003, 1.067)	0.032
Male	1.06 (0.63, 1.80)	0.816	#	#	#	#	#	#
Atrial fibrillation	3.10 (1.80, 5.35)	<0.001	1.39 (0.61, 3.18)	0.432	1.40 (0.61, 3.22)	0.432	1.44 (0.63, 3.27)	0.386
Myocardial infarction	1.88 (0.82, 4.33)	0.138	0.94 (0.25, 3.58)	0.926	0.94 (0.23, 3.80)	0.928	1.02 (0.26, 4.08)	0.977
Congestive heart failure	3.58 (1.76, 7.32)	<0.001	2.42 (0.80, 7.33)	0.118	2.08 (0.65, 6.65)	0.217	2.18 (0.68, 6.93)	0.189
Chronic obstructive pulmonary disease	2.72 (0.75, 9.91)	0.130	1.44 (0.20, 10.47)	0.716	1.12 (0.15, 8.51)	0.914	1.27 (0.17, 9.42)	0.813
Chronic kidney disease	2.31 (1.16, 4.61)	0.017	1.55 (0.53, 4.57)	0.800	1.90 (0.59, 6.15)	0.284	1.57 (0.50, 4.87)	0.436
History of malignancy	4.11 (1.00, 16.85)	0.050	2.64 (0.37, 18.73)	0.332	2.13 (0.28, 16.44)	0.467	2.61 (0.37, 18.23)	0.332
Swallowing dysfunction	24.06 (10.96, 52.81)	<0.001	11.04 (4.07, 29.94)	<0.001	11.04 (3.97, 30.67)	<0.001	13.42 (4.84, 37.23)	<0.001
Preexisting disability (mRS ≥ 2)	13.50 (4.67, 39.00)	<0.001	5.12 (1.17, 22.45)	0.031	4.06 (0.95, 17.43)	0.059	6.30 (1.47, 27.04)	0.013
Systolic blood pressure (per 10 mmHg increase)	1.08 (0.99, 1.18)	0.099	0.96 (0.81, 1.14)	0.666	0.96 (0.81, 1.14)	0.653	0.96 (0.81, 1.14)	0.661
Diastolic blood pressure (per 10 mmHg increase)	1.13 (0.99, 1.28)	0.078	1.14 (0.89, 1.47)	0.304	1.15 (0.89, 1.49)	0.275	1.19 (0.92, 1.54)	0.184
NIHSS at admission (per 1 unit increase)	1.25 (1.18, 1.33)	<0.001	1.11 (1.02, 1.21)	0.016	1.06 (0.97, 1.16)	0.199	1.07 (0.98, 1.17)	0.120
WBC (per 1,000 cell/mm^3^ increase)	1.16 (1.06, 1.27)	0.001	1.22 (1.05, 1.41)	0.009	1.19 (1.03, 1.37)	0.021	1.21 (1.04, 1.40)	0.012
NLR (per 1 unit increase)	1.10 (1.03, 1.19)	0.006	0.99 (0.91, 1.08)	0.788	0.97 (0.89, 1.06)	0.500	0.97 (0.89, 1.07)	0.582
Creatinine (per 1 mg/dL increase)	1.21 (0.90, 1.62)	0.203	#	#	#	#	#	#
FPG (per 10 mg/dL increase)	1.07 (1.01, 1.13)	0.019	0.99 (0.91, 1.08)	0.789	0.96 (0.87, 1.05)	0.357	0.97 (0.89, 1.06)	0.492

ASPECTS, Alberta Stroke Program Early CT Score; aOR, adjusted odds ratio; CI, confident interval; FPG, fasting plasma glucose; mRS, modified Rankin Scale; NA, not applicable; NIHSS, the National Institutes of Health Stroke Scale; NLR, neutrophil-to-lymphocyte count ratio; WBC, white blood cell count; #, not included. Hosmer-Lemeshow test was used (*P* = 0.644 for baseline ASPECTS model, *P* = 0.976 for 24-h ASPECTS model, and *P* = 0.962 for change in ASPECTS model).

We also explored the correlation between 24-h ASPECTS and predictive factors for SAP. There was a statistically significant negative correlation with NIHSS (r = −0.65, *p* < 0.001), FPG (r = −0.31, *p* < 0.001), absolute neutrophil count (ANC; r = −0.19, *p* = 0.001), WBC (r = −0.18, *p* = 0.001), age (r = −0.15, *p* = 0.005), and NLR (r = −0.12, *p* = 0.024; Supplementary Figure 2).

## 4 Discussion

This study focused on the relationship of infarction core using serial ASPECTS assessment and SAP in patients with thrombolyzed AACIS. Among these 3 scales, 24-h ASPECTS was shown to have better ability than both baseline ASPECTS and change in ASPECTS in predicting SAP suggesting that 24-h ASPECTS can serve as a valuable predictor of SAP. Our study observed a higher incidence rate of SAP (20.3%) compared to a previous study (14.3%) (23). The difference is possibly attributable to variations in study design, eligibility criteria, study timeframe, timing of clinician thresholds for antibiotic initiation, stroke severity, and diagnostic criteria for SAP between the two studies.

In our study, the AuROC of 24-h ASPECTS for predicting SAP was 0.84 (95% CI, 0.79–0.89). This differs from a previous study that evaluated the use of diffusion-weighted imaging-ASPECTS (DWI-ASPECTS) for SAP prediction (AuROC = 0.74 [95% CI, 0.68–0.80]) (24). Several factors may account for the differences. First, different neuroimaging modalities were used: our study utilized ASPECTS on NCCT, while the previous research employed DWI-ASPECTS. Second, the timing of ASPECTS assessment differed: we assessed follow-up NCCT-ASPECTS at 24 h, whereas the previous study assessed ASPECTS at the time of initial admission. Third, characteristics of the study populations differed: our study focused exclusively on patients with thrombolyzed AACIS, whereas the previous study also included patients with posterior circulation ischemic stroke. Our study found that the negative predictive value of 24-h ASPECTS was 95.2% (95% CI, 91.5–97.6). This implies that patients with 24-h ASPECTS greater than 6 might have a considerably lower risk for SAP. The sensitivity of baseline ASPECTS ≤8 for predicting SAP was 68.6% (95% CI, 56.4–79.1), which was lower than both the sensitivity of 24-h ASPECTS and change in ASPECTS. It is advisable, however, to use baseline ASPECTS with caution because of the challenges associated with performing brain NCCT during the early stages of stroke which can limit the detection of evolving ischemic patterns and related vasogenic and cytotoxic edema. Additionally, the initial assessment of NCCT-ASPECTS may be prone to higher error rates due to the presence of ambiguous early ischemic changes on baseline NCCT, requiring a high level of expertise to achieve accurate interpretation.

In our study, after adjusting for confounding factors in MVLR models, 24-h ASPECTS of ≤6 was significantly associated with SAP, demonstrating a greater ability to predict patients prone to SAP after IV-tPA compared to both baseline ASPECTS and change in ASPECTS. Our results indicate that the 24-h ASPECTS is more useful in predicting SAP following IV-tPA. In addition, we observed a significant negative correlation between high WBC, ANC, NLR, and 24-h ASPECTS. This finding could be attributed to increases in peripheral leukocyte counts which can elicit a sterile inflammatory response, resembling the immune response seen in early infections. The relationship between leukocyte counts, stroke outcomes, and infections was associated with ischemic brain injury-induced immune changes, leading to post-stroke infections and poorer outcomes in patients with AIS. Moreover, in experimentally-induced brain ischemia, infections have been shown to stimulate the autonomic nervous system and neuroendocrine pathways, leading to enhanced anti-inflammatory signaling. A robust cytokine-mediated anti-inflammatory response has recently been observed in stroke patients at a higher risk of infection (25).

Age, hyperglycemia, and NIHSS have been identified as important predictive factors for SAP (26). Blood glucose level has been shown to be correlated with cerebrovascular and inflammatory systems (27). In our study, we observed a significant negative correlation between 24-h ASPECTS and age, FPG, and NIHSS. This suggests a potential link between 24-h ASPECTS and SAP risk factors.

SAP typically develops within the first few days following stroke onset and is associated with adverse clinical outcomes, increased mortality, and higher healthcare costs (28). Currently, American Heart Association guidelines recommend NCCT as an imaging modality prior to IV-tPA. In Thailand, NCCT is more widely available than the more expensive magnetic resonance imaging. Thus, utilizing 24-h NCCT-ASPECTS, a cost-effective method which adds valuable information to other significant predictors, may provide more accurate identification of high-risk patients with SAP during the acute phase of AIS compared to using baseline ASPECTS and change in ASPECTS. This could also help achieve early identification of patients at risk for SAP and allow more timely implementation of preventive strategies such as systemic oral hygiene treatment, postural modification, and the swallowing screening test (29–32).

### 4.1 Strengths and limitations

The present study has some strengths. We used serial ASPECTS which is a simple and practical tool. Many of the demographics and comorbidities that were potential confounders that could have affected important determinants of outcomes of interest were adjusted for in MVLR thus achieving more accurate and reliable results. Multicollinearity is often the main obstacle that reduces the precision of estimated coefficients and thus weakening the statistical power of the regression model. Statistical significance of the comparison between SAP and non-SAP with collinearity was analyzed using AuROC. We also addressed the issue of collinearity among determinants by selecting the variable with the highest AuROC and incorporating it into the adjusted model to mitigate possible multicollinearity.

We acknowledge that our study has some limitations. First, this was a retrospective study with a limited number of patients, which might reduce the power of the study and increase statistical errors. Second, the single-center study design may have created a potential risk of publication bias. Third, in the study, changes in blood glucose levels during hospitalization were not recorded; only the FPG level was assessed. Fourth, this study did not include patients with brainstem stroke, which might be a potential risk factor of SAP, and studied only patients with AACIS receiving IV-tPA, which limits the generalizability of the results. Fifth, the requirement for mechanical ventilators is also an important issue highly correlated with SAP. We did not include mechanical ventilator requirement in MVLR for the following reasons: we aimed to study variables predictive of SAP occurrence within 24 h after IV-tPA administration, with the objective of early SAP prediction. Additionally, the data on mechanical ventilator requirement that we collected represents a consequence occurring after the onset of SAP. In future research, additional external validation is warranted to establish a more definitive conclusion. These findings are intriguing and have the potential to enhance SAP prediction performance, thereby facilitating surveillance and prompt treatment planning for patients at a higher risk of SAP. Exploring a novel clinical prediction model integrating NCCT 24-h ASPECTS with traditional factors for predicting SAP offers promising future research prospects.

## 5 Conclusion

24-h NCCT-ASPECTS showed better performance in SAP prediction compared to both baseline ASPECTS and change in ASPECTS. Furthermore, 24-h ASPECTS, employing a cut-off value of ≤6, demonstrated good predictive performance in predicting SAP. Our findings highlight the potential of 24-h ASPECTS as a promising and practical method for predicting SAP in patients with thrombolyzed AACIS.

## Data availability statement

The datasets used and analyzed during the current study are available from the corresponding author on reasonable request.

## Ethics statement

The studies involving human participants were reviewed and approved by the Human Research Ethics Committee at Saraburi Hospital (Certificate No. EC017/2566). Written informed consent from the patients/participants or patients/participants' legal guardian/next of kin was not required to participate in this study in accordance with the national legislation and the institutional requirements.

## Author contributions

SK: Conceptualization, Data curation, Formal analysis, Funding acquisition, Investigation, Methodology, Project administration, Resources, Software, Visualization, Writing – original draft, Writing – review & editing. AS: Supervision, Validation, Writing – review & editing. NA: Investigation, Writing – review & editing.

## Supplementary material

SUPPLEMENTARY FIGURE 1 Box-plots of serial Alberta stroke program early CT score by SAP. Differences in **(A)** baseline ASPECTS, **(B)** 24-hour ASPECTS, and **(C)** change in ASPECTS calculated by the Wilcoxon Rank Sum Test.

SUPPLEMENTARY FIGURE 2 Correlation between 24-hour ASPECTS and predictive factors of SAP (age, NIHSS, and FPG), and biomarkers of inflammatory response of patients with thrombolyzed AACIS.
