# Supplementary figures and images for: Serial ASPECTS to predict stroke-associated pneumonia after thrombolysis in patients with acute ischemic stroke
Source: Front Neurol. 2024 Apr 22;15:1364125. doi: 10.3389/fneur.2024.1364125 (PMC11071176; doi:10.3389/fneur.2024.1364125)

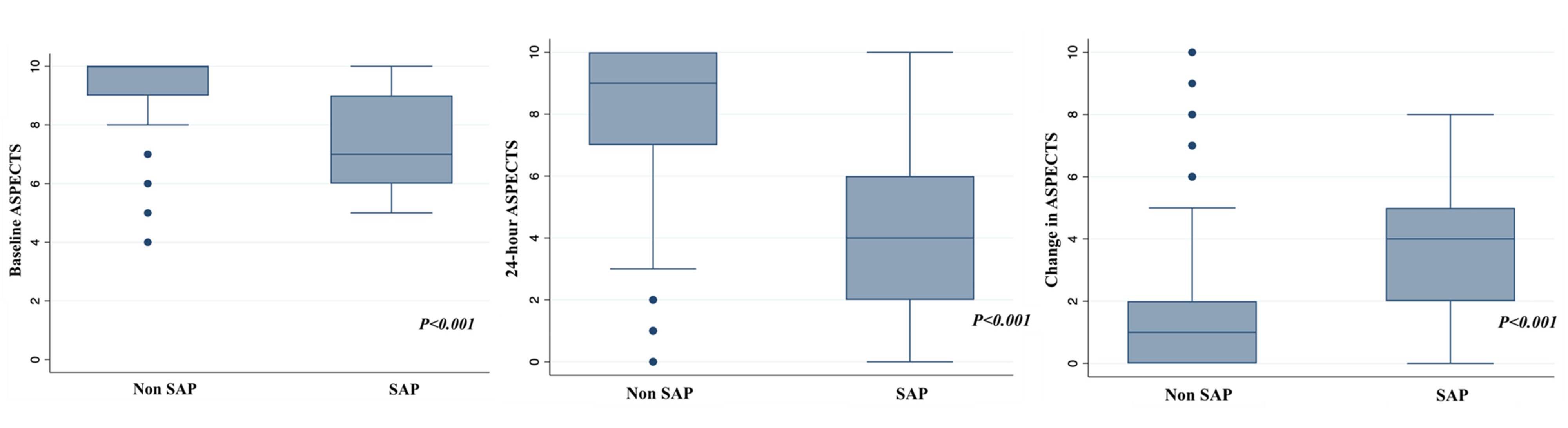

Supplement: SUPPLEMENTARY FIGURE 1 — Box-plots of serial Alberta stroke program early CT score by SAP. Differences in (A) baseline ASPECTS, (B) 24-hour ASPECTS, and (C) change in ASPECTS calculated by the Wilcoxon Rank Sum Test. [file Image_1.JPEG]

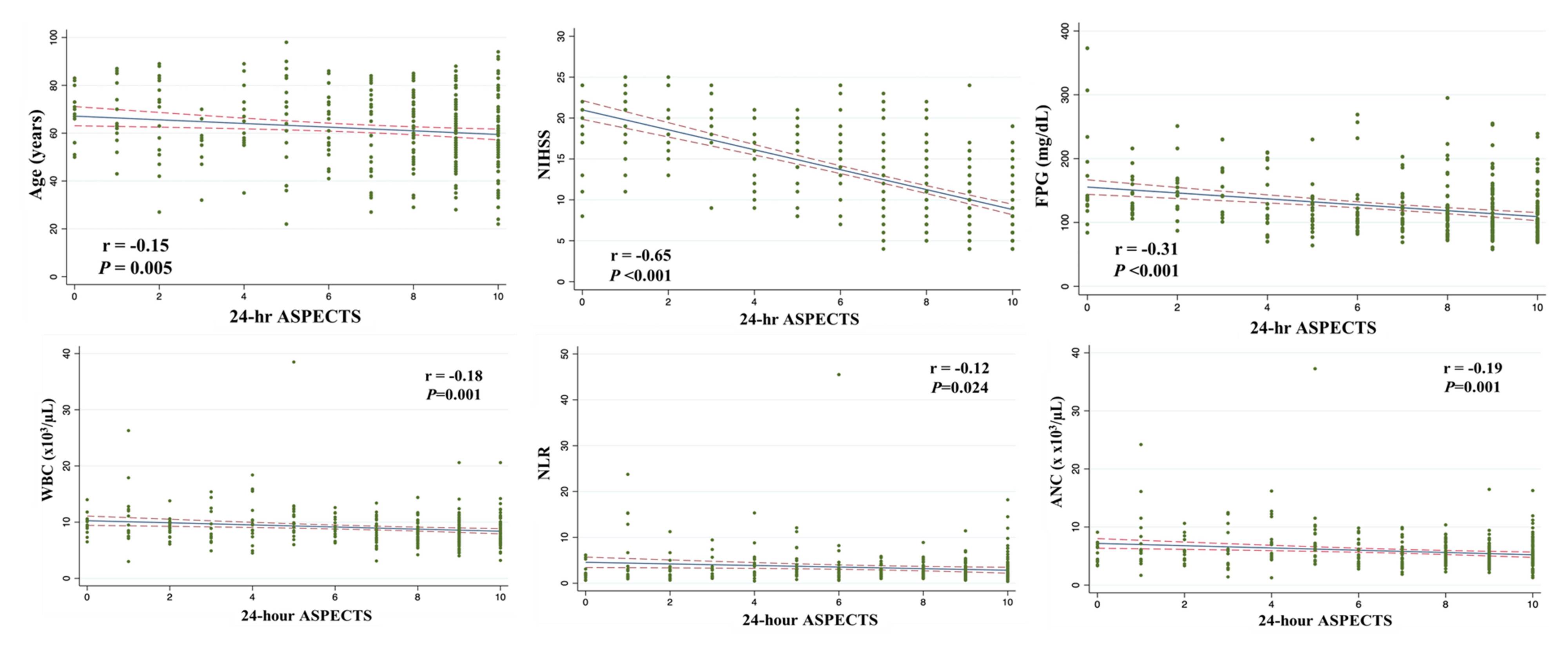

Supplement: SUPPLEMENTARY FIGURE 2 — Correlation between 24-hour ASPECTS and predictive factors of SAP (age, NIHSS, and FPG), and biomarkers of inflammatory response of patients with thrombolyzed AACIS. [file Image_2.JPEG]
