# Supplementary material for: Serial ASPECTS to predict stroke-associated pneumonia after thrombolysis in patients with acute ischemic stroke
Source: Front Neurol. 2024 Apr 22;15:1364125. doi: 10.3389/fneur.2024.1364125 (PMC11071176; doi:10.3389/fneur.2024.1364125)
Supplement: Supplementary file 3 [file Table_1.DOCX]

**Supplementary Table 1:** STROBE checklist

This protocol has been prepared in accordance with the 'Strengthening the Reporting of Observational Studies in Epidemiology' (STROBE) guidelines. We have completed the STROBE checklist provided below.

|  | Item No | Recommendation | Page No | Relevant text from manuscript |
| --- | --- | --- | --- | --- |
| **Title and abstract** | 1 | (*a*) Indicate the study’s design with a commonly used term in the title or the abstract | 2 | Lines 35 |
|  |  | (*b*) Provide in the abstract an informative and balanced summary of what was done and what was found | 2-3 | Lines 43-54 |
| Introduction | | | |  |
| Background/rationale | 2 | Explain the scientific background and rationale for the investigation being reported | 4-5 | Lines 68-104 |
| Objectives | 3 | State specific objectives, including any prespecified hypotheses | 5 | Lines 109-112 |
| Methods | | | |  |
| Study design | 4 | Present key elements of study design early in the paper | 6 | Lines 115-116 |
| Setting | 5 | Describe the setting, locations, and relevant dates, including periods of recruitment, exposure, follow-up, and data collection | 6,7 | Lines 115-116 and 143-153 |
| Participants | 6 | (*a*) Give the eligibility criteria, and the sources and methods of selection of participants. Describe methods of follow-up | 6 | Lines 126-135 |
|  |  | (*b*) For matched studies, give matching criteria and number of exposed and unexposed | NA | NA |
| Variables | 7 | Clearly define all outcomes, exposures, predictors, potential confounders, and effect modifiers. Give diagnostic criteria, if applicable | 9, 10 | Lines 190-192 and 208-212 |
| Data sources/ measurement | 8* | For each variable of interest, give sources of data and details of methods of assessment (measurement). Describe comparability of assessment methods if there is more than one group | 8-9 | Lines 164-186 |
| Bias | 9 | Describe any efforts to address potential sources of bias | 10 | Lines 208-210 |
| Study size | 10 | Explain how the study size was arrived at | 10 | Lines 222 |
| Quantitative variables | 11 | Explain how quantitative variables were handled in the analyses. If applicable, describe which groupings were chosen and why | 9 | Lines 197-202 |
| Statistical methods | 12 | (*a*) Describe all statistical methods, including those used to control for confounding | 10 | Lines 208 |
|  |  | (*b*) Describe any methods used to examine subgroups and interactions | NA | NA |
|  |  | (*c*) Explain how missing data were addressed | NA | NA |
|  |  | (*d*) If applicable, explain how loss to follow-up was addressed | NA | NA |
|  |  | (*e*) Describe any sensitivity analyses | NA | NA |
| Results | | |  |  |
| Participants | 13* | (a) Report numbers of individuals at each stage of study—eg numbers potentially eligible, examined for eligibility, confirmed eligible, included in the study, completing follow-up, and analysed | 10-11 | Lines 222-227, see Figure 1. |
|  |  | (b) Give reasons for non-participation at each stage | 10-11 | Lines 223-227, see Figure 1. |
|  |  | (c) Consider use of a flow diagram | 11 | see Figure 1. |
| Descriptive data | 14* | (a) Give characteristics of study participants (eg demographic, clinical, social) and information on exposures and potential confounders | 11-12 | Lines 228-251 |
|  |  | (b) Indicate number of participants with missing data for each variable of interest | NA | NA |
|  |  | (c) Summarise follow-up time (eg, average and total amount) | 11 | Lines 230 |
| Outcome data | 15* | Report numbers of outcome events or summary measures over time | 11 | 229 |

| Main results | 16 | (*a*) Give unadjusted estimates and, if applicable, confounder-adjusted estimates and their precision (eg, 95% confidence interval). Make clear which confounders were adjusted for and why they were included | 12-13 | Lines 264-271 |
| --- | --- | --- | --- | --- |
|  |  | (*b*) Report category boundaries when continuous variables were categorized | 11-12 | Lines 243-251 |
|  |  | (*c*) If relevant, consider translating estimates of relative risk into absolute risk for a meaningful time period | NA | NA |
| Other analyses | 17 | Report other analyses done—eg analyses of subgroups and interactions, and sensitivity analyses | 12  13 | - Lines 252-263 for ROC analysis and predictive value  - Line 272-276 for correlation analysis, see Supplementary Figure 2 |
| Discussion | | | |  |
| Key results | 18 | Summarise key results with reference to study objectives | 13 | Lines 278-282 |
| Limitations | 19 | Discuss limitations of the study, taking into account sources of potential bias or imprecision. Discuss both direction and magnitude of any potential bias | 16 | Lines 346-357 |
| Interpretation | 20 | Give a cautious overall interpretation of results considering objectives, limitations, multiplicity of analyses, results from similar studies, and other relevant evidence | 13-14  14 | Lines 286-295  Lines 296-302 |
| Generalisability | 21 | Discuss the generalisability (external validity) of the study results | 16 | Lines 350-352 |
| Other information | | | |  |
| Funding | 22 | Give the source of funding and the role of the funders for the present study and, if applicable, for the original study on which the present article is based | 19 | Line 397-400 |

*Give information separately for exposed and unexposed groups.

**Note:** An Explanation and Elaboration article discusses each checklist item and gives methodological background and published examples of transparent reporting. The STROBE checklist is best used in conjunction with this article (freely available on the Web sites of PLoS Medicine at http://www.plosmedicine.org/, Annals of Internal Medicine at http://www.annals.org/, and Epidemiology at http://www.epidem.com/). Information on the STROBE Initiative is available at http://www.strobe-statement.org.
